# Impact of selenium on the intestinal microbiome-eCBome axis in the context of diet-related metabolic health in mice

**DOI:** 10.3389/fimmu.2022.1028412

**Published:** 2022-11-11

**Authors:** Fredy Alexander Guevara Agudelo, Nadine Leblanc, Isabelle Bourdeau-Julien, Gabrielle St-Arnaud, Sébastien Lacroix, Cyril Martin, Nicolas Flamand, Alain Veilleux, Vincenzo Di Marzo, Frédéric Raymond

**Affiliations:** ^1^ Centre Nutrition, santé et société (NUTRISS), and Institute of Nutrition and Functional Foods (INAF), Université Laval, Québec, QC, Canada; ^2^ Canada Excellence Research Chair on the Microbiome – Endocannabinoidome Axis in Metabolic Health (CERC-MEND), Université Laval, Québec, QC, Canada; ^3^ Faculty of Agriculture and Food Sciences, Université Laval, Québec, QC, Canada; ^4^ Faculty of Medicine, Institut Universitaire de Cardiologie et Pneumologie de Quebec, Université Laval, Québec, QC, Canada

**Keywords:** microbiome, selenium, endocannabinoidome, inflammation, intestine, nutrition, metabolic health, endocannabinoid system

## Abstract

Dietary micronutrients act at the intestinal level, thereby influencing microbial communities, the host endocannabinoidome, and immune and anti-oxidative response. Selenium (Se) is a trace element with several health benefits. Indeed, Se plays an important role in the regulation of enzymes with antioxidative and anti-inflammatory activity as well as indicators of the level of oxidative stress, which, together with chronic low-grade inflammation, is associated to obesity. To understand how Se variations affect diet-related metabolic health, we fed female and male mice for 28 days with Se-depleted or Se-enriched diets combined with low- and high-fat/sucrose diets. We quantified the plasma and intestinal endocannabinoidome, profiled the gut microbiota, and measured intestinal gene expression related to the immune and the antioxidant responses in the intestinal microenvironment. Overall, we show that intestinal segment-specific microbiota alterations occur following high-fat or low-fat diets enriched or depleted in Se, concomitantly with modifications of circulating endocannabinoidome mediators and changes in cytokine and antioxidant enzyme expression. Specifically, Se enrichment was associated with increased circulating plasma levels of 2-docosahexaenoyl-glycerol (2-DHG), a mediator with putative beneficial actions on metabolism and inflammation. Others eCBome mediators also responded to the diets. Concomitantly, changes in gut microbiota were observed in Se-enriched diets following a high-fat diet, including an increase in the relative abundance of *Peptostreptococcaceae* and *Lactobacillaceae.* With respect to the intestinal immune response and anti-oxidative gene expression, we observed a decrease in the expression of proinflammatory genes *Il1β* and *Tnfα* in high-fat Se-enriched diets in caecum, while in ileum an increase in the expression levels of the antioxidant gene *Gpx4* was observed following Se depletion. The sex of the animal influenced the response to the diet of both the gut microbiota and endocannabinoid mediators. These results identify Se as a regulator of the gut microbiome and endocannabinoidome in conjunction with high-fat diet, and might be relevant to the development of new nutritional strategies to improve metabolic health and chronic low-grade inflammation associated to metabolic disorders.

## Introduction

Selenium (Se) is an essential micronutrient (1). It is absorbed from the diet through food or other forms of external supplementation, and its consumption is attributed important health benefits, including the improvement of immune response, fertility, and cognitive function (2). Se helps against oxidative stress and has attracted increasing attention to prevent diseases. There is abundant evidence linking low Se with the development of various chronic diseases, such as cardiovascular diseases (3, 4) and cancer, (5, 6).

Several recent studies have pointed to the possible role of dietary Se in the establishment of the intestinal microbiota and, subsequently, in the effects on host immunity and metabolism (7–9). Indeed, the diet is a key modulator of gut microbiota composition, and its imbalance may lead to the development of obesity (10, 11). Some studies have suggested that Se may inhibit adipocyte hypertrophy and adipogenesis (12, 13). Obesity is associated with a chronic low-grade inflammation state, and blood Se levels have been found to be inversely correlated with obesity, making Se deficiency a possible marker of excess adiposity (14, 15). Evidence from several populations suggests that elevated Se exposure may be associated with an adverse lipid profile (16–18), diabetes (19), and hypertension (20). Cross-sectional studies have shown that obesity is associated with low levels of serum Se and low intake of this mineral (21).

Specific circumstances that include genetic alterations, dietary changes and excessive stress, allow potentially pathogenic microorganisms to increase their growth, leading to a gut microbial imbalance known as dysbiosis (22). Se can modulate the composition of the microbiota and gut microbes can also affect Se levels in the host (23). There is strong evidence of a connection between obesity and gut microbiota dysbiosis, which will initiate and/or exacerbate inflammation *via* the phenomenon known as endotoxemia (24). Changes in the gut microbiome have been reported as a potential risk driver for obesity in humans (25–27). Biomarkers of Se nutritional status, including serum Se levels, as well as the activity of important selenoproteins, such as glutathione peroxidase (Gpx), may be associated with obesity (28, 29). Oxidative stress is found to be specifically increased in the adipose tissue in obesity, resulting in systemic oxidative stress that increases the risk of obesity-related metabolic complications. Therefore, down-regulation of Gpx protein expression may be associated with increased obesity-related systemic oxidative stress and the incidence of metabolic complications (30). Se-dependent glutathione peroxidases represent a family of enzymes which play an essential role in the reduction of lipid and hydrogen peroxides (31–33), and Se participates as the main redox component of this group of enzymes (34).

The expanded endocannabinoid (eCB) system, or endocannabinoidome (eCBome), comprises a vast network of receptors, their endogenous lipids ligands – which are chemically and biochemically related to the endocannabinoids - and the enzymes responsible for their synthesis and degradation (35). Endocannabinoids are long chain ω-6 polyunsaturated fatty acid (PUFA)-derived lipid mediators and include anandamide (AEA) and 2-arachidonoyl-glycerol (2-AG), mostly acting at G protein-coupled cannabinoid type 1 and type 2 (CB_1_ and CB_2_) receptors. Their respective congeners, the *N*-acyl-ethanolamines (NAEs) and 2-monoacyl-glycerols (2-MAGs), respectively, are important components of the eCBome, and, with the two eCBs, are involved in multiple physiological processes (36, 37). The recognized functions of eCBs include the regulation of satiety, energy control and lipid metabolism through the CB_1_ receptor, increasing food intake, reducing energy expenditure, and promoting fat accumulation in adipose tissue (38, 39). The eCBs acts both peripherally and centrally to stimulate metabolic processes that can lead to weight gain, and indeed obesity has been associated with eCB hyperactivity (40, 41). Conversely, other eCBome mediators, such as oleic acid- and palmitic acid-derived NAEs and 2-MAGs, counteract metabolic imbalance *via* their actions at other receptors (such as PPARα, GPR119 and TRPV1), and yet others, such as the ω-3 PUFA-derived ones, have been suggested to play anti-inflammatory actions *via* yet to be discovered receptors (42). Thus, the gradual development of obesity, and chronic low-grade inflammation therewith associated, following altered dietary habits might be due in part to changes in gut microbiota composition and imbalance of host eCBome signaling. Moreover, the consumption of essential micronutrients such as Se may influence not only the gut microbiota but also the way by which the precursors of eCB mediators are synthesized (43).

Given the role of Se in antioxidant activity, its implication in chronic low grade inflammation during obesity, and the impact it might have on changes in the composition and function of intestinal microbial communities, we investigated how: 1) dietary variations of Se in combination with high or low-fat diets impact intestinal microbial populations, and 2) the intake of this mineral alters the host eCBome-mediated response and immune status as well as antioxidant activity exerted by selenoproteins in the intestinal microenvironment. We speculated that variations in dietary Se concentrations would affect the gut-microbiota and its potential interaction with the host eCBome. Experiments were thus carried out with mice (males and females) to examine the impact of Se and diet formulation on the animals. We then stratified the data to assess the influence of sex on mouse responses to Se.

## Materials and methods

### Animals, diets and housing

The study was approved by the Université Laval animal ethics committee (CPAUL 2019-006). Forty-eight 6-week-old C57BL/6J male and female mice were purchased from Jackson Laboratory (USA) and were individually housed in the animal facility of the Institute of Nutrition and Functional Foods (INAF), in standard cages under controlled temperature (22°C) and relative humidity (50%) with a 12 h day/night cycle. At arrival, all mice were acclimated to their new environment for a one-week adaptation period, during which they received a normal chow diet (AIN-93G-purified diet #110700, Dyets Inc., Bethlehem, PA, USA). Following this time, they were randomly assigned to 4 groups (n=12 per group, 6 males and 6 females). The groups were defined according to 4 diet designs. **Table S1** presents the formulation for the four diet groups set as follows: Enriched (1 mg/kg) and depleted (0.1 mg/kg) concentrations of Se in combination with High Fat-High Sucrose (HFHS: 23.6% fat, 17% sucrose, LabDiet, St.Louis, MO, USA), and Low Fat-Low Sucrose (LFLS: 4.3% fat, 7% sucrose, LabDiet, St.Louis, MO, USA). The diets were formulated to be isonitrogenous, but different in terms of lipids and energy between the HFHS and LFLS diets. Total energy in diets was determined with an adiabatic Parr 6300 calorimeter (Parr Instrument Company, Moline, IL, USA) and was similar among LFLS and among HFHS diets (Se-depleted LFLS 4010.75 cal/g; Se-enriched LFLS 4008.55 cal/g; Se-depleted HFHS 4913.05 cal/g; Se-enriched HFHS 4937.45 cal/g). Dietary protein content was determined by combustion (Duma’s method) using a LECO FP‐528 apparatus (LECO Corporation, St. Joseph, MI, USA) and was also found similar between the diets (Se-depleted LFLS 14.99% [w/w] and 599 cal/g; Se-enriched LFLS 14.85% [w/w] and 594 cal/g; Se-depleted HFHS 18.92% [w/w] and 757 cal/g; Se-enriched HFHS 18.54% [w/w] and 742 cal/g). Dietary fat content was measured with an ANKOMXT10 Extractor (ANKOM Technology, Macedon, NY, USA) and was different between the diets, reflecting the fact that we have low-fat and high-fat diet (Se-depleted LFLS 3.74% [w/w] and 337 cal/g; Se-enriched LFLS 2.6% [w/w] and 234 cal/g; Se-depleted HFHS 22.93% [w/w] and 2064 cal/g; Se-enriched HFHS 22.21% [w/w] and 1999 cal/g). Animals were fed *ad libitum* with these diets for 28 days and had access to *ad libitum* water for the same period of time. Body weight and food intake were monitored twice weekly. Mice were sacrificed by cardiac puncture. Whole blood was collected in K3-EDTA tubes to obtain plasma (1,780 × g, 10 min). Ileum and caecum tissues were collected at 10 cm and 2 cm from the ileocecal junction, respectively. Luminal contents were collected in PBS with gentle scraping. Tissue samples from both ileum and caecum were treated with RNAlater Stabilization Solution (ThermoFisher, USA) to preserve the integrity of RNA until its subsequent extraction. All samples were stored at −80°C until further analysis.

### Endocannabinoidome mediators

Lipids were extracted from plasma (40 μL) samples as in (44). In brief, plasma samples were diluted to a volume 500ul with Tris 50mM. 5 μL of deuterated standards were added to each sample then vortexed. Two milliliter of toluene was added and samples were vortexed for 30 seconds. Samples were then placed dry ice-ethanol bath to allow freeze the aqueous phase. Ileum and caecum samples (5 to 10 mg) were extracted exactly as in (45). The organic phases were then collected and evaporated under a stream of nitrogen. Lipid extracts were then resuspended with 50 μL of mobile phases (50% Solvent A and 50% solvent B) then injected on the injected onto an HPLC column (Kinetex C8, 150 × 2.1 mm, 2. 6 μM; Phenomenex) as described before (46). Quantification of eCBome-related mediators was performed using a Shimadzu 8050 triple quadrupole mass spectrometer. The following metabolites were quantified: 1/2-arachidonoylglycerol (AG), 1/2-DHG), 1/2-docosapentaenoyl(n-3)-glycerol (2-DPG), 1/2-eicosapentaenoyl-glycerol (2-EPG), 1/2-linoleoyl-glycerol (LG), 1/2-oleoyl-glycerol (2-OG), arachidonic acid (AA), docosahexaenoic acid (DHA), docosaepentaenoic acid (DPA), eicosapentaenoic acid (EPA), stearidonic acid (SDA), linoleic acid (LA), *N*-arachidonoyl-ethanolamine, (AEA), *N*-docosahexaenoyl-ethanolamine (DHEA), *N*-linoleoyl-ethanolamine (LEA), *N*-oleoyl-ethanolamine (OEA), *N*-palmitoyl-ethanolamine (PEA), *N*-stearoylethanolamine (SEA), prostaglandin E_2_ (PGE_2_), *N*-Palmitoyl-Glycine and *N*-Oleoyl-Serotonin. prostaglandin D_2_ (PGD_2_), prostaglandin E_1_ (PGE_1_), prostaglandin E_2_ (PGE_2_), prostaglandin E_3_ (PGE_3_), 1a,1b-dihomo prostaglandin F2α (1a,1b-dihomo PGF2α), thromboxane B2 (TBX2) and *N*-docosapentaenoyl-ethanolamine (DPEA).

### 16S rRNA gene sequencing

Intestinal luminal contents were lysed using bead beating (0.1-mm silica beads). Samples were lysed with the OMNI Bead Ruptor 12 (Precellys) for 2 cycles of 1 minute. Before enzymatic digestion with 50 mg of lysozyme and 200 U/μL mutanolysin (37°C, 45 min). Microbial DNA was extracted using the QIAamp DNA Stool minikit (Qiagen, CA, USA), and amplification of the V3-V4 region was performed using the primers Bact-0341-b-S-17 (5′-CCTACGGGNGGCWGCAG-3′) and Bact-0785-a-A-21 (5′-GACTACHVGGGTATCTAATCC-3′) (Illumina, CA, USA). Libraries were purified using magnetic beads AMPURE XP (Beckman Coulter Canada Lp), and libraries were assessed on gel using QIAexcel (Qiagen, CA, USA). High-throughput sequencing (2- by 300-bp paired end) was performed on a MiSeq platform (Illumina, CA, USA). The average reads per samples was 12705, with a minimum of 2344 reads and a maximum of 31309 reads. Sequences were processed using the DADA2 package (version 1.16.0) (47) and associations with bacterial taxa were obtained using the Ribosomal Database Project reference database release Silva version 132. Microbiome abundances were normalized using rarefaction (Rarefaction; Vegan R package). Reads were rarefied to 5,000 reads to account for depth bias (48). Samples with read count lower than 5000 but higher than 2000 reads were kept in the analysis as is. Prior to rarefaction, we observed 5113 ASV and after rarefaction we observed 4923 ASV. The raw sequences were deposited to SRA under accession PRJNA886990.

### mRNA isolation, reverse transcription and qPCR

RNA was extracted from the ileum and caecum samples with the RNeasy Plus mini kit (Qiagen, CA, USA) according to the manufacturer’s instructions and eluted in 30 μL of UltraPure distilled water (Invitrogen, USA). RNA concentration and purity were determined by measuring the absorbance of RNA in a nanodrop at 260 nm and 280 nm. A total of 500 nanograms of RNA was reverse transcribed with a high-capacity cDNA reverse transcription kit (Applied Biosystems, CA, USA). We used 7500 Real-Time PCR System (Applied biosystems, CA, USA) to perform quantitative PCR to assess the expression of 4 eukaryotic genes related with inflammation (*Il-10*, *Tgfβ-1*, *Il1β* and *Tnfα*) and 3 antioxidant activity genes (*Gpx1*, *Gpx2* and *Gpx4*) with one housekeeping gene (*Hprt*). Primers and probes for TaqMan qPCR assays were purchased as commercial kits (ThermoFisher Scientific, Burlington, ON, Canada) and TaqMan assay IDs were as follows: *Hprt* (Mm03024075_m1), *Il-10* (Mm01288386_m1), *Tgfβ-1* (Mm01178820_m1), *Il1β* (Mm00434228_m1), *Tnfα* (Mm00443258_m1), *Gpx1* (Mm00656767_g1), *Gpx2* (Mm00850074_g1) and *Gpx4* (Mm04411498_m1). All expression data were normalized by the threshold cycle (2^-ΔΔCT^) method using *Hprt* as internal control (49).

### Statistical analyses

Data are expressed as mean ± SEM. Ranked mixed linear regressions (nlme R package) and two-way analysis of variance (ANOVA) followed by test of contrast were used to identify significant intestinal segment or Se effects and interactions. The eCBome mediator responses to the diet were investigated using ANOVA linear contrast *post hoc* analysis. Generalized linear regression models were used to identify the effects of Se and interactions with fat, and sex. We used a three-way ANOVA based on a linear model that included interactions between diet formulation (LFLS vs HFHS), Se concentration (depleted vs enriched) and sex of the animal (female or male). The differences were considered statistically significant with P values of P<0.05 using contrast test between enriched and depleted Se levels, LFLS and HFHS formulations, the sex of animals (female and male) and the combination between Se levels and formulations. Associations between microbiome families and eCBome mediators were investigated by building a correlation network. Spearman correlations were performed between metataxonomic, lipidomics and quantitative PCR results. Variables were scaled and centered prior to correlation analysis. Adjustments for multiple testing were obtained using false-discovery rate (FDR) and correlation were included in the network if their FDR corrected p-values were < 0.05. Correlation network was built using Cytoscape version 3.9.1. Node positions were manually adjusted to improve readability. Analyses were performed with R software version 4.0.2. Principal-component analysis was performed using the FactoMineR version 2.6 R package (49). PERMANOVA was performed between two of the segments of intestine (ileum and caecum) with 999 permutations in conjunction with Canberra distances between samples using package vegan in R (v2.5.7).

## Results

### Low dietary selenium intake impairs weight gain with low fat low sucrose diet

**Figure 1:**
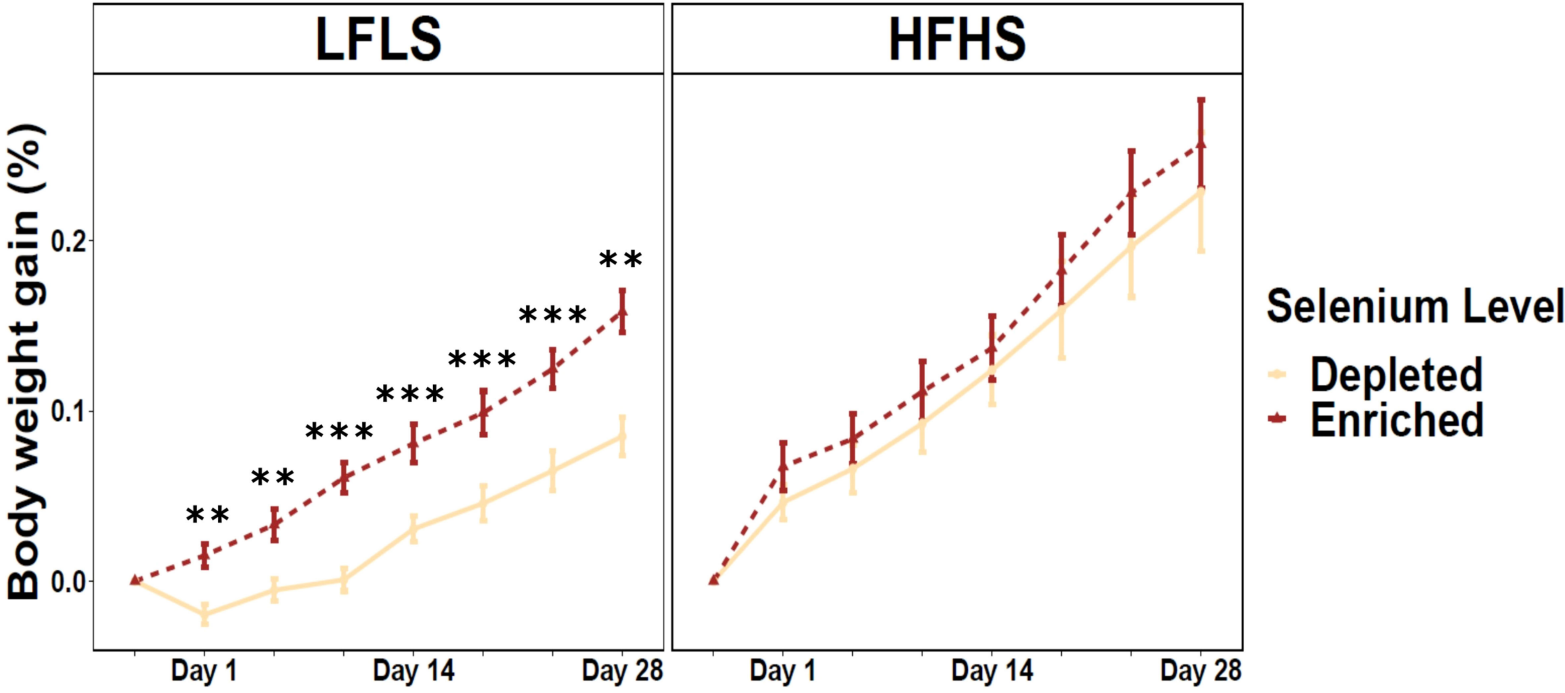
Weight gain in mice fed Se-enriched and Se-depleted LFLS or HFHS diets. Groups of 12 mice (6F/6M) were fed Se-enriched and/or Se-depleted diets combined with LFLS or HFHS diet for 28 days. Generalized linear regression models were used to identify the effects of time or Se and interactions. Data are expressed as mean ± SEM (n = 12). *P* values of linear contrast analysis are identified when significant ‘***’ P < 0.001, ‘**’, P < 0.01, using contrast between enriched and depleted Se levels, LFLS and HFHS formulations and the combination between Se levels and formulations.

Mice fed 28 days of Se-enriched diets showed an increased weight gain compared to mice that received Se-depleted diets, both in the HFHS and LFLS diets. Strikingly, in LFLS this increase was relatively stronger and statistically significant after 28 days (p=0.0005) and at all measured time points (**Figure 1**). Notably, a slight weight loss was observed in the first days of the study with the Se-depleted LFLS diets, which did not occur with the other diets. Although higher in Se-enriched formulation, the body weight of mice with HFHS showed no statistically significant difference between Se-enrichment and Se-depletion during the protocol (p=0.56). Male mice showed higher weight gain than females for both Se-enriched and Se-depleted diets, accompanied by high-fat and low-fat formulations (**Figure S1**). Energy intake was not significantly different between Se-depleted and Se-enriched diets for both HFHS (p=0.16) and LFLS (p=0.91), suggesting that the difference in weight gain was not associated with energy consumption. Nonetheless, energy intake was significantly higher in HFHS compared to LFLS diet (p<0.0001). These results suggest that Se impact on weight gain interacts with diet composition, but that it might not be linked with differences in energy intake.

### Dietary Selenium impacts intestinal immune and antioxidant gene expression

**Figure 2:**
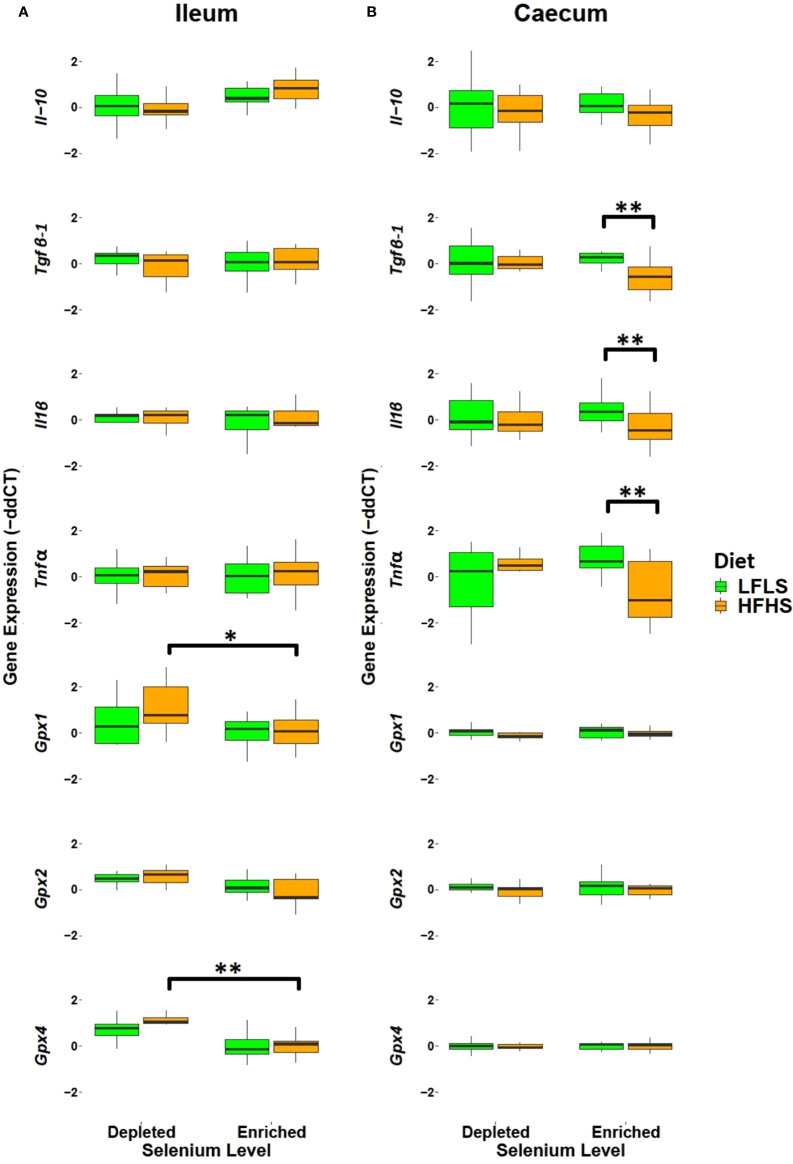
Intestinal mRNA expression of immune response and antioxidant state genes as fold change (FC) calculated using the ΔΔ*C_T_* method. **(A)** Ileum. **(B)** Caecum. Gene expression was normalized to *Hprt*. *P* values of linear contrast analysis are detailed when significant ‘**’, P < 0.01, ‘*’, P<0.05 using contrast test between enriched and depleted Se levels, LFLS and HFHS formulations and the combination between Se levels and formulations. The samples were analysed at day 28 of the study.

We examined how the intestinal inflammatory response and antioxidant activity adapted to variations in dietary Se. In the ileum, dietary Se did not significantly modulate immune-related genes (**Figure 2A**). In the caecum, expression of *Tgfβ-1* (p=0.017), *Il1β* (p=0.035), and *Tnfα* (p=0.0151) were significantly reduced in HFHS diet with enriched Se (**Figure 2B**). Reduction of *Tnfα* and *Il1β* expression in caecum after Se supplementation could potentially mediate a counteraction of HFHS diet-induced caecum inflammation. With respect to gene expression level of Gpx family, we observed a general increase in the mRNA levels, which was statistically significant for *Gpx4* (p=0.00005) and *Gpx1* (p=0.022*)* in the ileum after the HFHS diet with depleted Se. No overall significant impact of diet on Gpx gene expression was observed in the caecum. The *Gpx4* gene showed a higher level in males between high and low fat with Se-depleted diets (p=0.013). Higher antioxidant Gpx gene expression in HFHS diet with depleted Se could serve to compensate for the reduced presence of Se. No overall significant impact of diet on Gpx gene expression was observed in the caecum. Overall, these results point to a potential anti-inflammatory effect of Se enrichment in the context of a HFHS diet.

### Selenium modulates circulating 2-MAGs and intestinal fatty acid amides and prostaglandins

**Figure 3:**
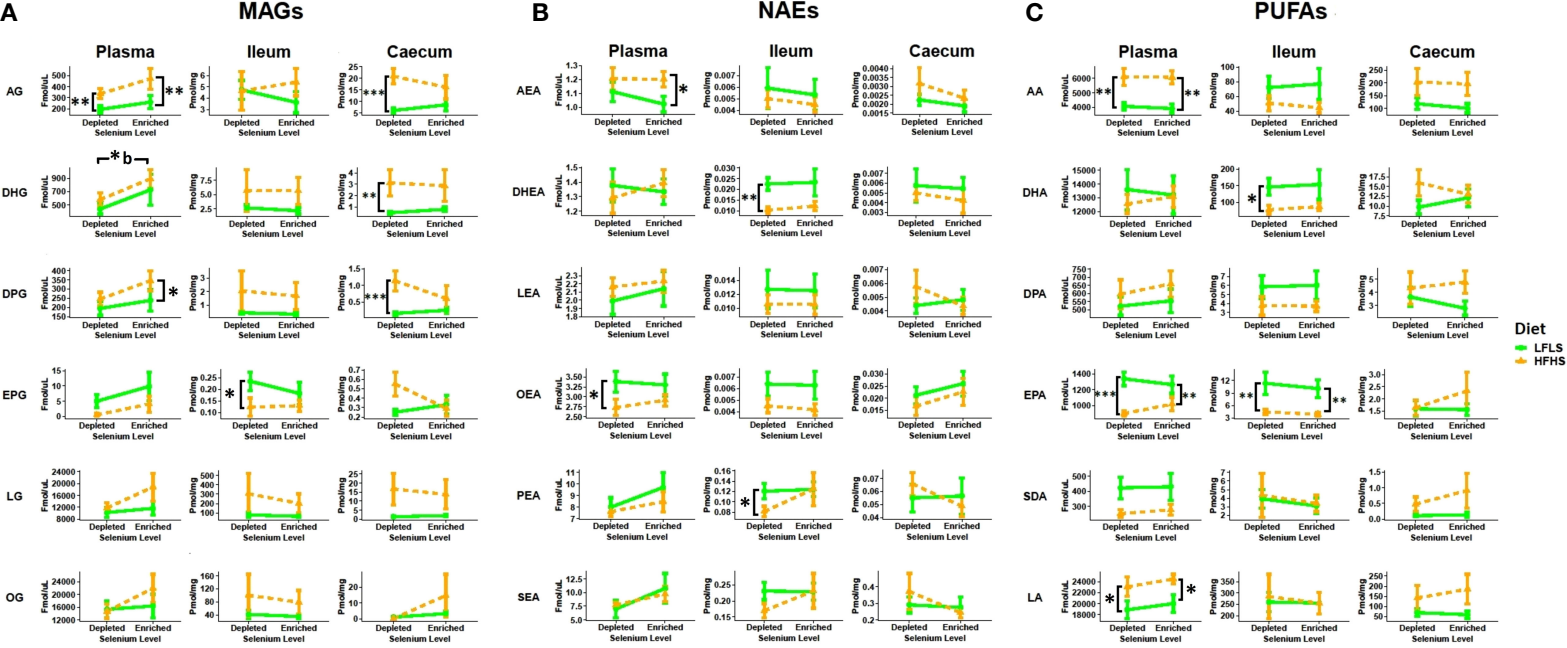
Diet and Se modulation of the endocannabinoidome and fatty acid precursors. **A**) 2-monoacylglycerols (2-MAGs), **B**) *N*-acylethanolamines (NAEs) and **C**) long chain ω-6 and ω-3 polyunsaturated fatty acid (PUFA) response to Se-enriched and Se-depleted LFLS or HFHS diets. Line chart representation of the eCBome mediators in Plasma, Ileum and Caecum. Data are expressed as the mean ± SEM (n = 12). *P* values of linear contrast analysis are detailed when significant ‘***’ P < 0.001, ‘**’, P < 0.01, ‘*’, P<0.05 using contrast test between enriched and depleted Se levels, LFLS and HFHS formulations and the combination between Se levels and formulations. Letter b indicates difference between HFHS conditions. The samples were analysed at day 28 of the study.

Lipid mediators, including the eCBome, can play a key role in inflammation. Thus, we evaluated the eCBome mediators (MAGs and NAEs) and their fatty acid precursors (PUFAs) in plasma, ileum and caecum samples (**Figure 3**). The eCBome response was different between plasma and the two intestinal segments studied. Moreover, modulation by diet and Se intake were not consistent between tissues. In plasma, we observed that Se-enriched diets enhanced the levels of circulating 2-MAGs, particularly in HFHS diets. Indeed, 2-DHG showed a significant increase associated with the enrichment of Se in the diets (**Figure 2A**). 2-AG, 2-DPG and 2-EPG showed a similar behavior, although the differences were not statistically significant. Circulating levels of NAEs and PUFAs were not associated with Se intake, but were influenced by fat and sugar content of diets (**Figures 3B, C**). As expected, circulating levels of AEA were higher with the HFHS diet (50), while OEA levels were higher with the LFLS diet. AA was increased with the HFHS diet, while EPA showed an increase with the LFLS diet. In the intestine, a statistically significant modulation was only associated with dietary fat and sugar levels rather than Se variations. In the ileum, 2-EPG, DHEA, PEA, AA, DPA, and EPA were higher with the LFLS than the HFHS diet. By contrast, in the caecum, 2-AG, 2-DHG and 2-DPG levels were higher with the HFHS than the LFLS diet.

**Figure 4:**
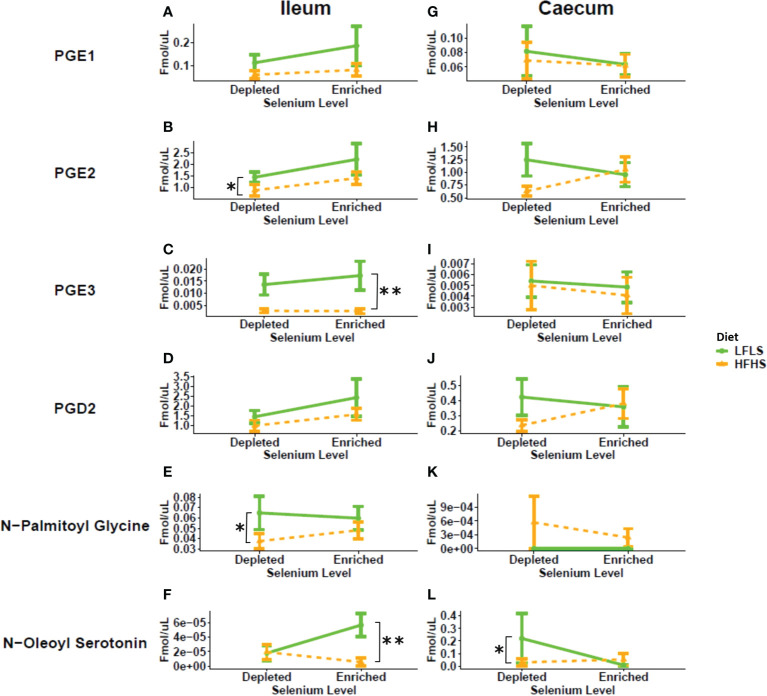
Response of prostaglandins and additional eCBome mediators in Se-enriched and Se-depleted LFLS or HFHS diets in the intestine. Line chart representation of the eCBome mediators in Ileum **(A-F)** and Caecum **(G-L)**. Data are expressed as the mean ± SEM (n = 12). *P* values of linear contrast analysis are detailed when significant ‘**’, P < 0.01, ‘*’, P<0.05 using contrast test between enriched and depleted Se levels, LFLS and HFHS formulations and the combination between Se levels and formulations. The samples were analysed at day 28 of the study.

In the ileum, Se enrichment in the diet also reversed the reduction in the levels of *N*-palmitoyl-glycine caused by HFHS, and resulted in increased levels of *N*-oleoyl-serotonin under LFLS (**Figure 4**). In the caecum, *N*-oleoyl-serotonin were higher under a HFHS diet only in the absence of Se. *N*-palmitoyl-glycine and *N*-oleoyl-serotonin have been proposed to play anti-inflammatory effects *via* different receptors, i.e., agonism of GPR18 and PPARα or TRPV1, in the case of the former mediator, and antagonism of TRPV1 and inhibition of endocannabinoid inactivation by fatty acid amide hydrolase (FAAH), in the case of the latter (51, 52). Thus, Se supplementation either increased the levels of these mediators under conditions of HFHS, in the ileum, or abolished their HFHS-induced increases, in the caecum, suggesting in both case an anti-inflammatory effect of this mineral, whereas the Se-induced elevation of ileal *N*-oleoyl-serotonin levels in LFLS mice may be linked to increased body weight in these mice.

In addition to endocannabinoid congeners and PUFAs, we also quantified other lipid mediators that could respond differentially to dietary changes of Se (**Figure 4**). In this sense, we evaluated the response of PGD_2_, PGE_1_, PGE_2_ and PGE_3_ (**Figure 4**). In some tissues, PUFAs acting as precursors of prostaglandins can be produced from the hydrolysis of the corresponding polyunsaturated MAGs (53). We observed a Se-dependent increase for both PGE_2_ and PGE_3_ in the ileum of the HFHS diet accompanied by Se enrichment, which reflected the changes observed in this tissue of: 1) AA and EPA, and 2) EPG, but not 2-AG, possibly suggesting a role as prostaglandin biosynthetic precursors only for PUFAs and not necessarily for polyunsaturated 2-MAGs.

In summary, Se intake affected the concentrations of circulating PUFAs, 2-MAGs and of other eCB-like mediators as well as of PGE_2_ and PGE_3_ mostly in the ileum. Modulation of the eCBome and prostaglandins by Se and diet could play a role in the chronic low-grade inflammation of mice under a HFHS diet.

### Segment-specific switch of intestinal microbiome populations during selenium feeding

**Figure 5:**
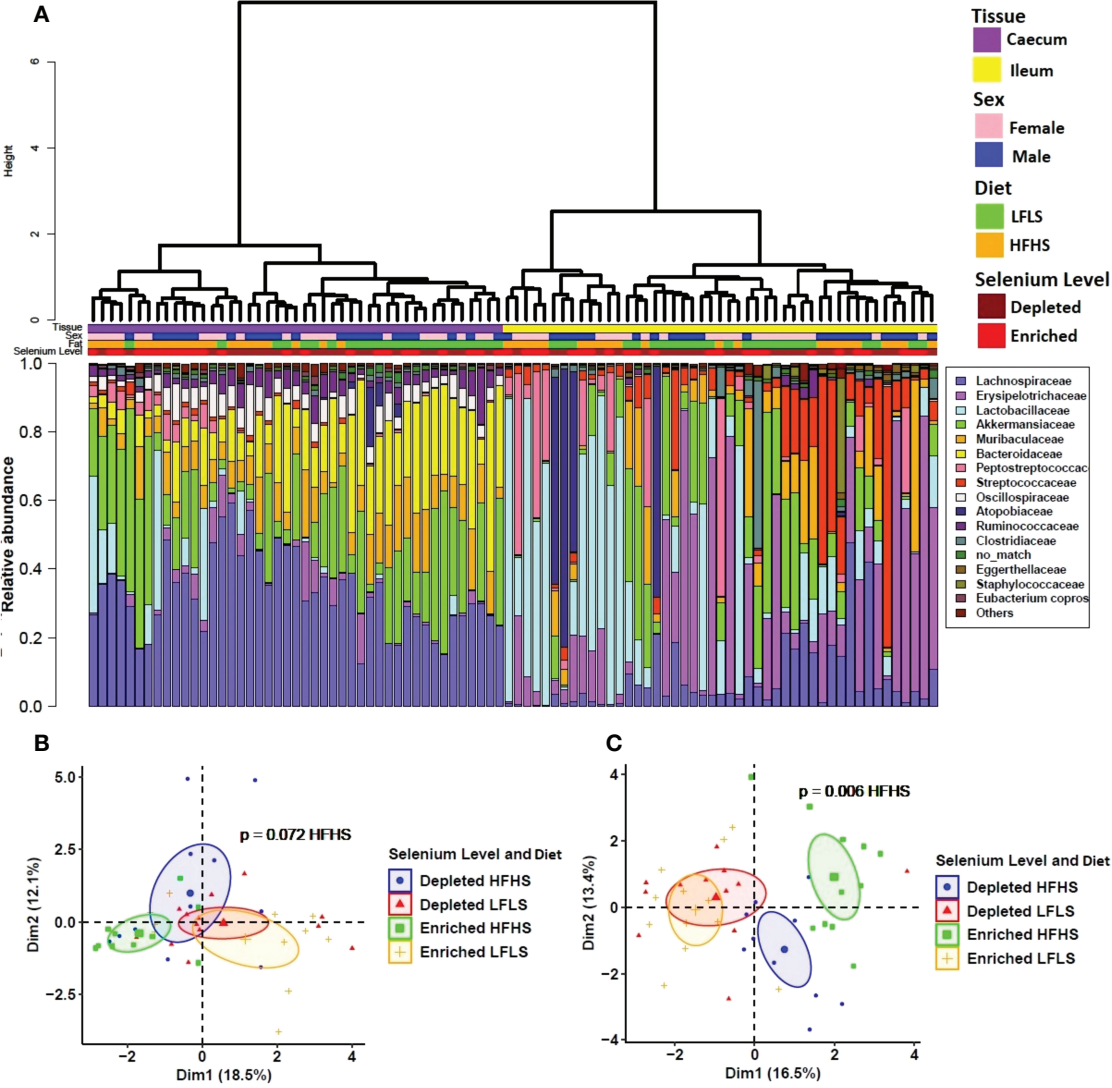
Intestinal microbiota composition in response to Se-enriched and Se-depleted LFLS or HFHS diets. **A**) Relative bacterial abundance at the family rank in response to Se-enriched and Se-depleted LFLS or HFHS diets in the intestinal segments ileum and caecum. Families representing less than 1% of total bacterial abundance were aggregated. Dendrogram showing hierarchical clustering based on Canberra distance between samples is showed and determines the sample ordering. The corresponding annotations for tissue, sex, diet and Se level are displayed. **B**) Principal component analysis shows the impact of depleted/enriched concentrations of Se with High and Low-Fat Sucrose diets on gut microbiota composition of the ileum and **C**) in the caecum. Permanova indicates significance of microbiota composition differences between the dietary conditions. The samples were analysed at day 28 of the study.

**Figure 6:**
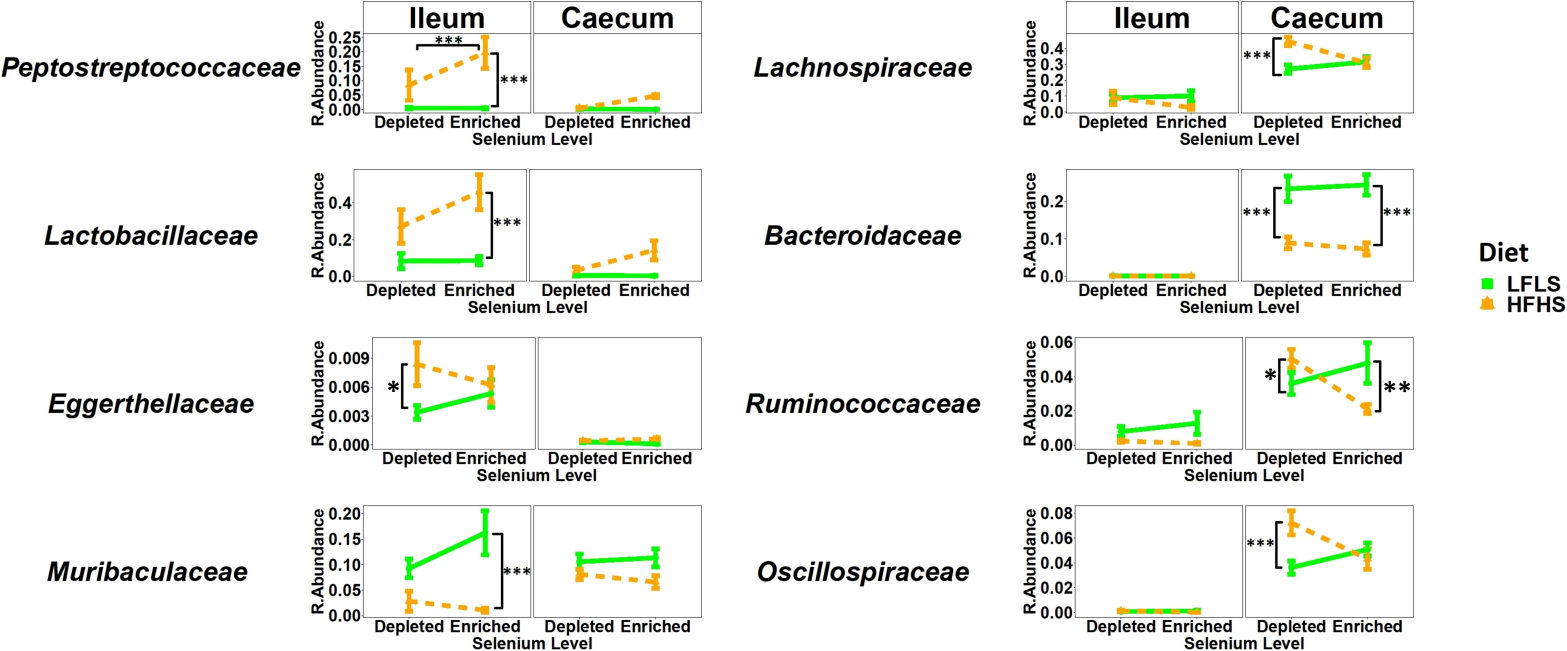
Relative bacterial abundance at the family rank in response to Se-enriched and Se-depleted LFLS or HFHS diets in the intestinal segments ileum and caecum. Data are expressed as the mean ± SEM (n = 12). *P* values of linear contrast analysis are detailed when significant ‘***’ P < 0.001, ‘**’, P < 0.01, ‘*’, P<0.05 using contrast test between enriched and depleted Se levels, LFLS and HFHS formulations and the combination between Se levels and formulations. The samples were analysed at day 28 of the study.

We investigated whether gut microbial taxa responded differentially to dietary variations of Se and whether these putative associations remained independent of dietary fat and sucrose intake. The structure of the intestinal microbiota shows a strong differentiation between the ileum and caecum segments (P<0.01, PERMANOVA) (**Figure 5A**). Interindividual differences were higher in ileum than in caecum and the two segments reacted differently to the dietary treatments. Principal component analysis shows that the effect of Se combined with both diets was limited in the ileum (**Figure 5B**) while Se intake had a significant impact on the caecum microbiota in mice fed with HFHS diet but not with LFLS diet (**Figure 5C**). Detailed results for the statistical analysis of bacterial families in relation with intestinal segment, selenium intake, diet composition and the sex of the animal are shown in **Table S2**. Modulation of taxa between the segments was similar for several bacterial families, although some modulated taxa were detected in only one of the two segments studied (**Figure 6**). For instance, *Lactobacillaceae* and *Peptostreptococcaceae* were higher with the HFHS diet and were further increased by Se supplementation for both segments. Moreover, these two taxa were of low abundance or not detected in mice under a LFLS diet and in mice under a depleted Se HFHS diet. Dietary changes in Se allowed an increase of the relative abundance in some species over others according to intestinal location. The *Eggerthellaceae* family was only detected in the ileum under Se depletion conditions, and its relative abundance in this segment was also higher with the HFHS than the LFLS diet. Likewise, the *Muribaculaceae* family showed a higher relative abundance in the ileum only with HFHS Se-enriched diets. Concomitantly, the *Bacteroidaceae, Ruminococcaceae* and *Oscillospiraceae* families were mostly detected in the caecum. In this intestinal segment, Se intake affected the relative abundance of *Oscillospiraceae* and *Ruminococcaceae* in interaction with the diet. Indeed, *Oscillospiraceae* was increased by the HFHS diet in Se depleted conditions. *Bacteroidaceae* was higher with the LFLS than HFHS diet in the caecum. Thus, although the general diet composition in macronutrients was the main driver of microbiota composition in both intestinal segments, Se intake did influence specific families of bacteria, especially in combination with the HFHS diet.

### Sex influences the impact of selenium on the intestine

In addition to studying the effects of dietary variations in Se and fat formulation, we were also interested in studying how the sex of the animals affected the phenotype of the mice (**Figures S1**-**S5**). Sex of the mice had limited impact on the expression level of genes associated to the inflammatory profile and the intestinal anti-oxidative state (**Figure S2**). However, we observed that *Gpx4* presented superior levels in males fed with Se-enriched diets accompanied with LFLS compared to females.

Sex differences in the endocannabinoids have already been demonstrated (54, 55). Circulating 2-MAGs such as 2-AG, 2-DHG and 2-LG showed higher levels in females on Se-enriched diets accompanied by the HFHS diet, while 2-DPG was higher only in males with the same regimen of diets (**Figure S3**). Regarding circulating NAEs, we observed that LEA was increased in males with both diets, and OEA was higher in males only with the LFLS diet (**Figure S4**). Similarly, DPA showed elevated levels in males with both diets, while SDA showed higher levels in males only with the LFLS diet (**Figure S5**). At the intestinal level, particularly in the ileum, mediators such as 2-DPG and 2-EPG showed higher levels in males than in females. In the caecum, we observed elevated responses of 2-AG, 2-DHG, 2-DPG, 2-EPG and 2-LG in males with Se enriched and HFHS diets. Ileum DHEA levels were higher in males on Se-enriched accompanied with LFLS, although not significantly different from females. By contrast, the mediator PEA showed higher levels only in females on Se-depleted and LFLS diets. In the caecum, the fatty acids AA, DPA and SDA were favored by Se-enriched-HFHS diets since they showed elevated levels under these conditions only in males, while DHA showed higher levels in females with depleted Se and HFHS diet. Overall, the sex of the animals was an important factor in modulating the eCBome in response to the diet and Se levels, and, *vice versa*. Dietary factors were important in determining the observation of sex differences in eCBome mediators.

Se level shifts with HFHS diets modulated microbial families also in a sex-dependent manner (**Figure S6**). In females, *Peptostreptococcacea* and *Lactobacillaceae* exhibited an increase in their relative abundance under Se-depletion with HFHS diets. In males, microbial families such as *Eggerthellaceae*, *Streptococcaceae* and *Oscillospiraceae* showed an increase with Se-enriched diets also accompanied with HFHS (**Figure S6**). This points to a differential role of Se and its participation in shaping the intestinal microbiota according to the sex of the host.

### Overview of intestinal microbiome-eCBome axis in response to Se and diet

In the previous sections, we have focused on the influence of Se intake on intestinal gene expression, microbiome composition and lipid mediator concentrations. However, diet composition, including fat content, and the sex of the mice were major factors in modulating numerous variables (**Table S2**). Overall, Se intake influenced 19 variables; diet composition 52 variables; and sex 35 variables, with numerous interactions between factors. This general approach to data interpretation indicates that the diet is the main driver of the biological processes we investigated, with many variables affected by the sex of the mice. Nevertheless, Se remains a dietary factor that can modulate the microbiome of the caecum in addition to mostly modulating 2-MAGs and inflammation related-genes.

**Figure 7:**
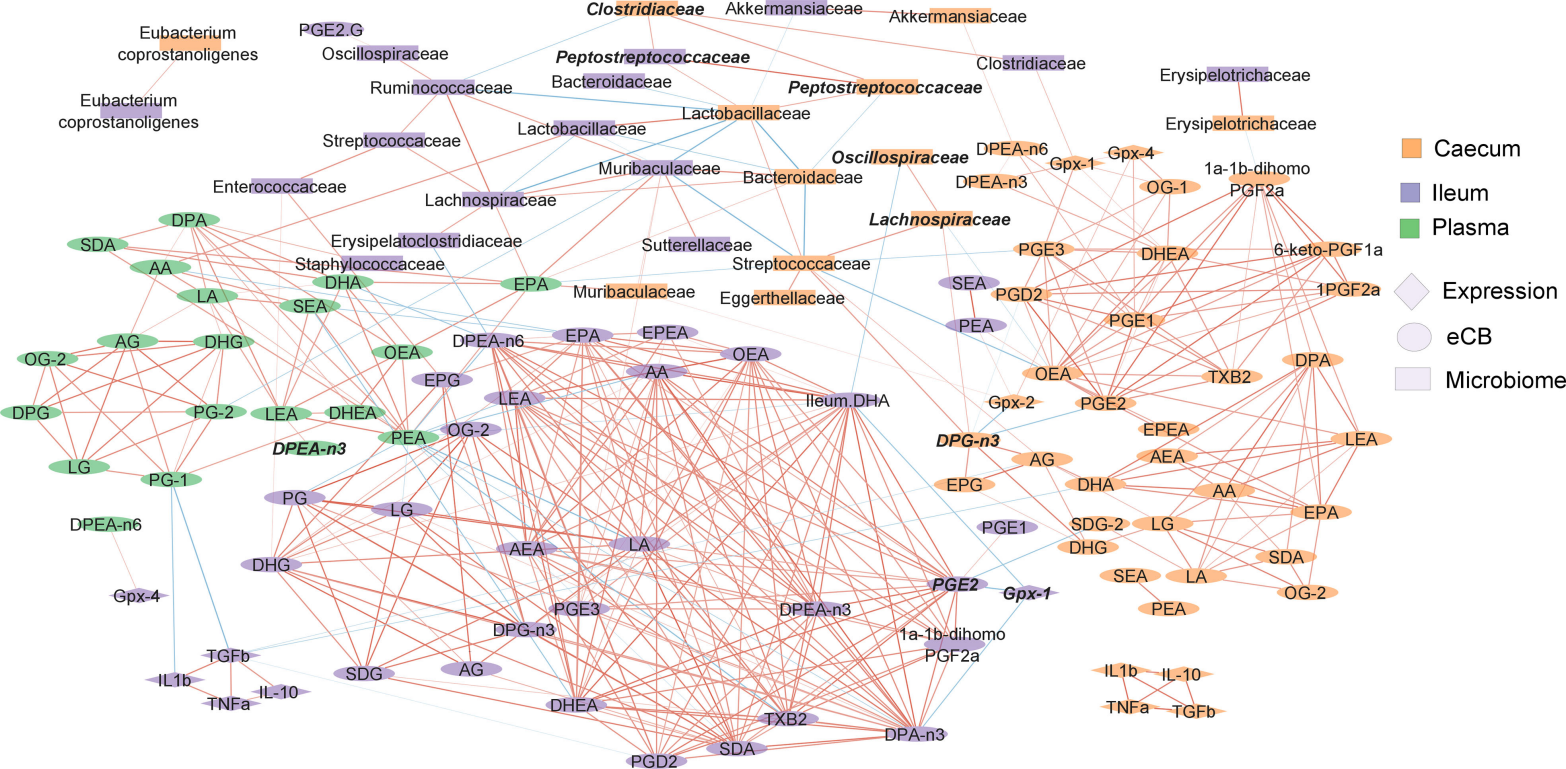
Correlation network showing the relationship between the endocannabinoidome, the gut microbiome and intestinal gene expression of immune and Gpx family genes. Color of the nodes indicate the tissue. Shape of the nodes indicate the type of data. The color of edges represents positive (red) or negative (blue) correlation. Width of the edges are proportional to the strength of the correlation. Features in bold italics were statistically significant between the depleted and enriched Se HFHS diet. The position of nodes was manually adjusted to improve clarity.

Our observations show that many variables seem linked in their responses to experimental factors. We constructed a correlation network to investigate these relationships (**Figure 7**). Endocannabinoidome mediators and prostaglandins clustered by tissue. Molecules did not show inter-tissue correlation with themselves. Nonetheless, 2-MAGs generally correlated together within each tissue, as did NAEs, and prostaglandins. Limited correlation was observed between the two major families of eCBome mediators. Several taxa were positively correlated between the two studied intestinal segments, including *Akkermansiaceae, Erysipelotrichaceae, Lactobacillaceae*, *Muribaculaceae*, and *Peptostreptococcaceae.* Most correlations of the gut microbiota with lipid mediators were with fatty acids such as AA, EPA or DPEA. Still, *Enterococcaceae* in the ileum were positively associated with 2-MAGs, specifically 2-EPG and 2-DHG.

To better understand the influence of Se in response to the HFHS diet, we investigated the correlation of variables that were significantly different between the Se-depleted and Se-enriched HFHS diets (**Figure 7**, in bold italics). In the ileum, *Gpx1* gene expression and PGE_2_ concentration were inversely correlated. Relative abundance of *Peptostreptococcaceae* in both ileum and caecum were positively correlated with *Clostridiaceae*, as were *Lachnospiraceae* and *Oscillospiraceae* in the caecum. It is of note that these four taxa belong to the order *Eubacteriales.*

## Discussion

The present study assessed the impact of Se supplementation and depletion in combination with LFLS and HFHS formulations on Gpx selenoprotein expression, intestinal inflammatory markers, and, particularly, eCBome signaling and gut-microbiota composition. This was a short duration study and although our aim was not to induce systemic and tissue inflammation, previous studies have shown that 4 weeks of an obesogenic diet in mice are sufficient to alter the inflammatory phenotype and induce changes in the gut microbiota (56). In general, we observed that mice consuming Se-enriched food gained more weight in comparison with Se-depleted diets, which is in accordance with previous studies (57–59). Interestingly, Se-induced weight gain was higher under a LFLS compared to a HFHS diet, an interaction that could potentially explain disagreements in the literature on the effects of this mineral on weight gain (60–62). In both cases, the impact of Se-related weight gain did not seem associated with energy intake. Modulation of Gpx gene expression in the ileum was also influenced by the interaction between the diet and Se, as was cytokine gene expression in the caecum, although not in the same dietary conditions. In addition, the impact of Se intake on: 1) plasma 2-MAGs, particularly the proposed anti-inflammatory compound 2-DHG, 2) intestinal eCB-like molecules (i.e fatty acid amides) with targets that mitigate inflammation, and 3) pro- and anti-inflammatory prostaglandins, was also influenced by the accompanying diet. These results support the hypothesis of a potential anti-inflammatory effect of Se consumption in response to the diet and exerted also through these alterations of the eCBome. On the other hand, the observed increase of *Gpx1* and *Gpx4* expression in the ileum following the depleted Se LFLS diet might represent a compensatory mechanism to enhance antioxidant activity despite lower Se availability (63). Finally, caecum gene expression of *Tnfα*, *Il1β* and *Tgfβ-1* was only modulated by Se HFHS diet, as were most of the microbiome taxa found here to be influenced in the ileum and the caecum. The impact of Se intake on microbiota composition, both in ileum and caecum, suggests that the effect of this mineral on microbiome-related factors could be strongly linked with the diet and, potentially, with the inflammatory state of the intestine. Our results also support the hypothesis that micronutrients can affect the host and microbiome response to a HFHS diet. Taken together, these results demonstrate that the impact of Se on the microbiome is diet-dependent and associated with the inflammatory state of the caecum. Further studies are needed to clarify whether the changes in gut microbiota composition observed here following Se supplementation may contribute to its suggested anti-inflammatory actions.

In order to evaluate whether the enrichment with Se would have an impact not only on weight gain but also on eCBome signaling (64), we evaluated the impact that dietary variations of this mineral would have on the levels of circulating and intestinal eCBome mediators (MAGs and NAEs) and their corresponding PUFAs. Se-enriched diets showed a significant effect on MAGs more than NAEs and PUFAs. Particularly, the circulating mediators 2-AG, 2-DHG and 2-DPG showed increased concentrations with an enriched Se diet, with slight differences between male and female mice. These mediators have been implicated in metabolic activity and brain function (37, 65, 66). The fact that we have identified circulating 2-MAGs with activity in the brain is consistent with Se proposed involvement in cognitive development (67). Indeed, human health can be improved by increasing dietary intake of Se and ω-3 PUFAs (68). However, dietary enrichment or depletion of Se had little impact on the production of eCBome mediators at the intestinal level, which instead were sensitive to the caloric and macronutrient composition of the diet in agreement with previous studies (69). Indeed, mediators such as 2-AG, 2-DHG, 2-DPG and 2-LG showed a significant increase only in the presence of HFHS and exclusively in the caecum. An exception to this rule is represented by intestinal *N*-palmitoyl-glycine and/or *N*-oleoyl-serotonin, two eCBome mediators that modulate the activity of targets (GPR18 or PPARα and TRPV1, respectively) involved in immune development and inflammation (51, 52, 70), which were modulated by Se in a diet-dependent manner. Interestingly, *N-*oleoyl-serotonin, which has also been shown to reduce intestinal GLP-1 release (71), was the only variable significantly modulated, and increased, by Se in the LFLS diet. The inhibitory action on GLP-1 of *N-*oleoyl-serotonin, as well as its antagonism of TRPV1 and inhibition of FAAH, may all underlie the increased body weight observed in LFLS mice following Se supplementation, since these three molecular targets have all been associated with lower body weight (72, 73). However, future studies are required in order to investigate this hypothesis.

The fact that dietary fat levels play a key role in the production of intestinal 2-MAGs could suggest a bivalent effect of dietary fatty acids on the production of some mediators, depending on intestinal location. In fact, it has previously been shown that the level of dietary fat also affects the abundance of gut microorganisms in a segment-specific manner (50). Regarding the gut microbiota, dietary variations of Se were found to differentially impact specific microbial families according to intestinal location. Microbial families such as *Peptostreptococcaceae* and *Lactobacillaceae* were favored by Se-enrichment in the diet as they showed a higher relative abundance, while other intestinal families such as *Eggerthellaceae*, and *Oscillospiraceae* showed a significant reduction in their growth. This suggests possible affinity of some microbial species for this mineral. Variations in its concentration could act as an inorganic modeling agent in microbial communities. Looking at how Se variations affect the same microbial species in different intestinal locations, we observed that those microbial families that presented relatively high relative abundances in ileum were instead less represented in the caecum, and *vice versa* (**Figure 6**). Se absorption occurs in the proximal sections of the small intestine (74), and this fact as well as the presence of molecular mechanisms for Se acquisition by some microbial species, could explain the enhancement for some microbial species over others. The interaction between Se variations and dietary fat/sucrose produced an interesting effect on the growth of *Ruminococcaceae* (**Figure 6**), since Se-enriched diets in combination with HFHS reduced the growth of this microbial species, whereas a low-fat formulation boosted its growth. This may suggest that depending on the amounts of some macronutrients, the appropriate levels of micronutrients may promote and/or adversely impact specific microbial targets. Similarly, the presence/absence of high fat and sucrose in the diet participated in the modulation of some microbial families, as previously shown in previous studies (75). Previous studies have shown sex differences for both endocannabinoid mediators and microbial communities under different conditions (dietary fat and sucrose) (54, 55, 69). However, to the best of our knowledge, this is the first study to report differences under conditions of dietary variations of Se for both systems. Our results show that males exhibited superior levels for some mediators over females, such as LEA, OEA, 2-DPG, among others. Similarly at the intestinal level, specific microbial families responded differentially in a sex-dependent manner. In general, the observed sex-differences were diet-dependent.

## Conclusions

Overall, our results indicate that Se intake modulates intestinal response to diet in mice. On one side, Se enrichment contributed to weight gain when mice were fed with LFLS diet. On the other hand, Se enrichment in mice consuming HFHS diet favored the presence of eCBome mediators, prostaglandins and the expression of genes involved in inflammation regulation. We also showed that the microbiome of the ileum and the caecum responded to Se intake in a diet dependent manner. Although the microbiota of the two intestinal segments shared some taxa modulation, their microbial ecology is different. In conclusion, our findings show that Se might, in combination with the diet, modulate intestinal processes and contribute to a healthy response to dietary stress induced by the HFHS diet. This study shows how complex dietary components may interact with the gut-microbiota ecosystem and the host metabolism. The present findings should open the way for mechanistic studies investigating the molecular basis of the impact of micronutrients on the microbiome-eCBome axis, in response to Se deficit or supplementation, and the role of this interaction in low grade inflammation such as that accompanying diet-induced obesity.

## Data availability statement

The data presented in the study are deposited in the SRA repository, accession number PRJNA886990.

## Ethics statement

The animal study was reviewed and approved by Université Laval animal ethics committee (CPAUL 2019-006).

## Author contributions

FG, NL and FR conceived and designed the work. FG, NL, GS-A, SL and CM carried out the experiments FG, IB-J, SL, NF, FR, and VD contributed to data analysis and interpretation. FG, FR, AV, and VD drafted the manuscript. All authors contributed to the article and approved the submitted version.

## Funding

This work was carried out within the activities of the Canada Excellence Research Chair in Microbiome-Endocannabinoidome Axis in Metabolic Health, held by V. Di Marzo and funded by the Canadian Federal Government Tri-Agency (CERC programme) and the CFI Leaders fund. FR is funded by NSERC Discovery Grant (RGPIN-2020-03922). FR and VD are funded by CIHR (Team Grant: Canadian Microbiome Initiative 2: Research Teams – Dissecting host-microbiome modifiers of type 2 diabetes risk and complications). Computing was performed on Compute Canada infrastructure (FR, RRG2734). This work was also supported by the Sentinelle Nord program of Université Laval (Canada First Research Excellence Fund) via its support to the International Mixed Unit - MicroMenu between the Italian National Research Council (CNR) and Université Laval (VD).

## Acknowledgments

Special thanks to Annie Pelletier, at the time with the Department of animal science, who assisted us in the animal protocol.

## Conflict of interest

The authors declare that the research was conducted in the absence of any commercial or financial relationships that could be construed as a potential conflict of interest.

## Publisher’s note

All claims expressed in this article are solely those of the authors and do not necessarily represent those of their affiliated organizations, or those of the publisher, the editors and the reviewers. Any product that may be evaluated in this article, or claim that may be made by its manufacturer, is not guaranteed or endorsed by the publisher.

## Supplementary material

Supplementary Figure 1 Weight gain in Female and Male mice fed with fed Se-enriched and Se-depleted LFLS or HFHS diets. Groups of 12 mice (6F/6M) were fed Se-enriched and/or Se-depleted diets for 28 days. Generalized linear regression models were used to identify the effects of time or Se and interactions. Data are expressed as mean ± SEM (n = 6). *P* values of linear contrast analysis are detailed in the bottom when significant ‘**’, P < 0.01, ‘*’, P<0.05 using contrast test between enriched and depleted Se levels, LFLS and HFHS formulations and the combination between Se levels and formulations.

Supplementary Figure 2 Intestinal mRNA expression of immune response and anti-oxidant state genes as fold change (FC) calculated using the ΔΔ*C_T_* method by sex. **(A)** Ileum. **(B)** Caecum. Gene expression was normalized to *Hprt*. *P* values of linear contrast analysis are detailed when significant ‘**’, P < 0.01, ‘*’, P<0.05 using contrast test between enriched and depleted Se levels, LFLS and HFHS formulations and the combination between Se levels and formulations. The samples were analysed at day 28 of the study.

Supplementary Figure 3 Diet and Se modulation of 2-monoacylglycerols (2-MAGs) by sex. Line chart representation of the 2-MAGs in Plasma **(A-F)** Ileum **(G-L)** and Caecum **(M-R)**. Data are expressed as the mean ± SEM (n = 6). *P* values of linear contrast analysis are detailed in the bottom when significant ‘**’, P < 0.01, ‘*’, P<0.05 using contrast test between enriched and depleted Se levels, LFLS and HFHS fat formulation and the combination between Se levels and formulations. Letter b indicates difference between HFHS conditions. The samples were analysed at day 28 of the study.

Supplementary Figure 4 Diet and Se modulation of *N*-acylethanolamines (NAEs) by sex. Line chart representation of the NAEs in Plasma **(A-F)** Ileum **(G-L)** and Caecum **(M-R)**. Data are expressed as the mean ± SEM (n = 6). *P* values of linear contrast analysis are detailed in the bottom when significant ‘**’, P < 0.01, ‘*’, P<0.05 using contrast test between enriched and depleted Se levels, LFLS and HFHS fat formulation and the combination between Se levels and formulations. Letter a indicates difference between LFLS conditions. The samples were analysed at day 28 of the study.

Supplementary Figure 5 Diet and Se modulation of precursors of endocannabinoids fatty acids (PUFAs) by sex. Line chart representation of the PUFAS in Plasma **(A-F)** Ileum **(G-L)** and Caecum **(M-R)**. Data are expressed as the mean ± SEM (n = 48). *P* values of linear contrast analysis are detailed in the bottom when significant ‘***’ P < 0.001, ‘**’, P < 0.01, ‘*’, P<0.05 using contrast test between enriched and depleted Se levels, LFLS and HFHS formulations and the combination between Se levels and formulations. Letter a indicates significance between LFLS conditions. The samples were analysed at day 28 of the study.

Supplementary Figure 6 Diet and Se modulation of intestinal microbiome by sex. Line chart representation of the Relative abundance in Ileum and Caecum. Data are expressed as the mean ± SEM (n = 48). *P* values of linear contrast analysis are detailed in the bottom when significant ‘***’ P < 0.001, ‘**’, P < 0.01, ‘*’, P<0.05 using contrast test between enriched and depleted Se levels, LFLS and HFHS formulations and the combination between Se levels and formulations. Letter b indicates significance between HFHS conditions. The samples were analysed at day 28 of the study.
