# Supplementary figures and images for: Impact of selenium on the intestinal microbiome-eCBome axis in the context of diet-related metabolic health in mice
Source: Front Immunol. 2022 Nov 11;13:1028412. doi: 10.3389/fimmu.2022.1028412 (PMC9692131; doi:10.3389/fimmu.2022.1028412)

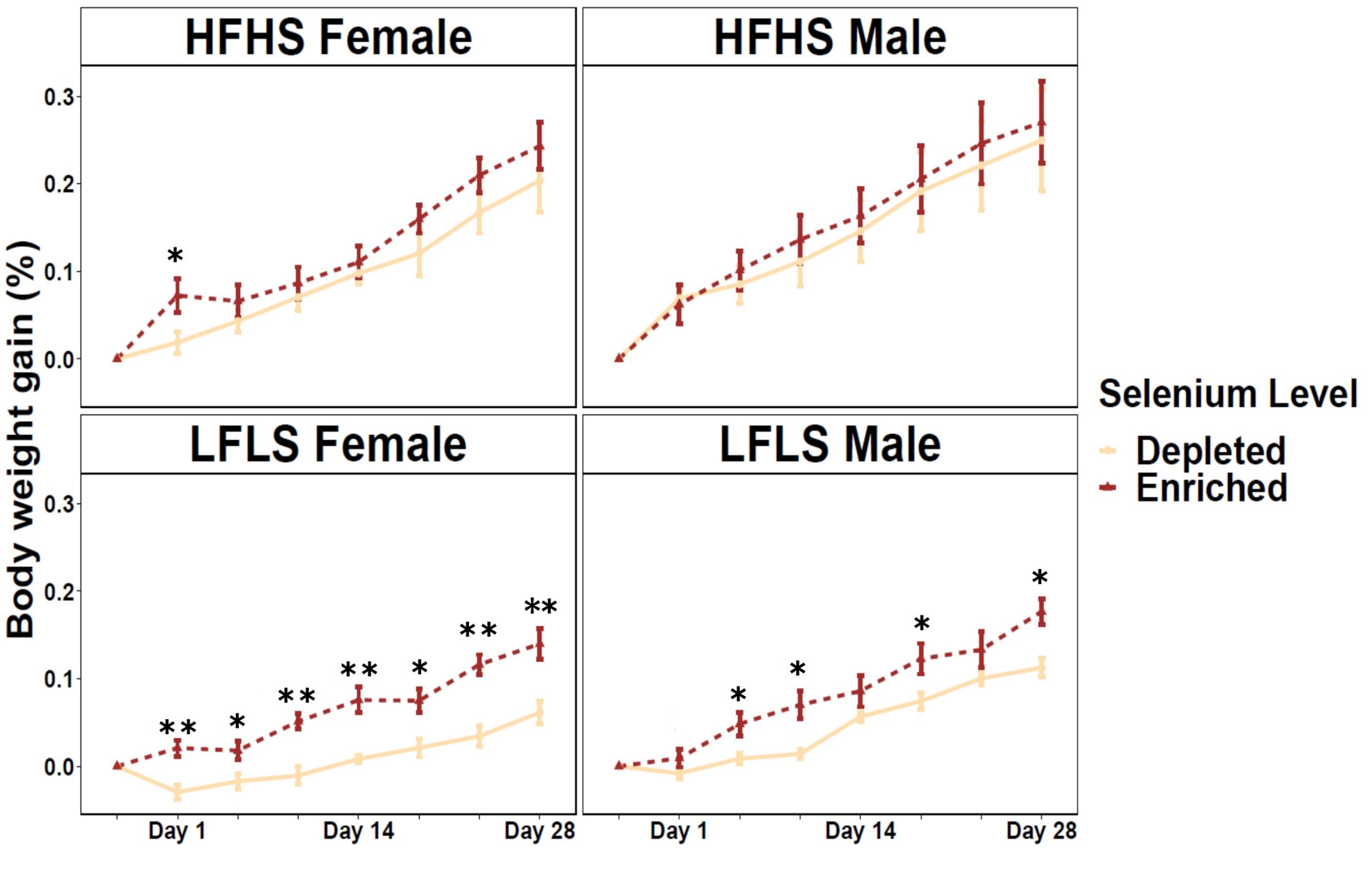

Supplement: Supplementary Figure 1 — Weight gain in Female and Male mice fed with fed Se-enriched and Se-depleted LFLS or HFHS diets. Groups of 12 mice (6F/6M) were fed Se-enriched and/or Se-depleted diets for 28 days. Generalized linear regression models were used to identify the effects of time or Se and interactions. Data are expressed as mean ± SEM (n = 6). P values of linear contrast analysis are detailed in the bottom when significant ‘**’, P < 0.01, ‘*’, P<0.05 using contrast test between enriched and depleted Se levels, LFLS and HFHS formulations and the combination between Se levels and formulations. [file Image_1.jpeg]

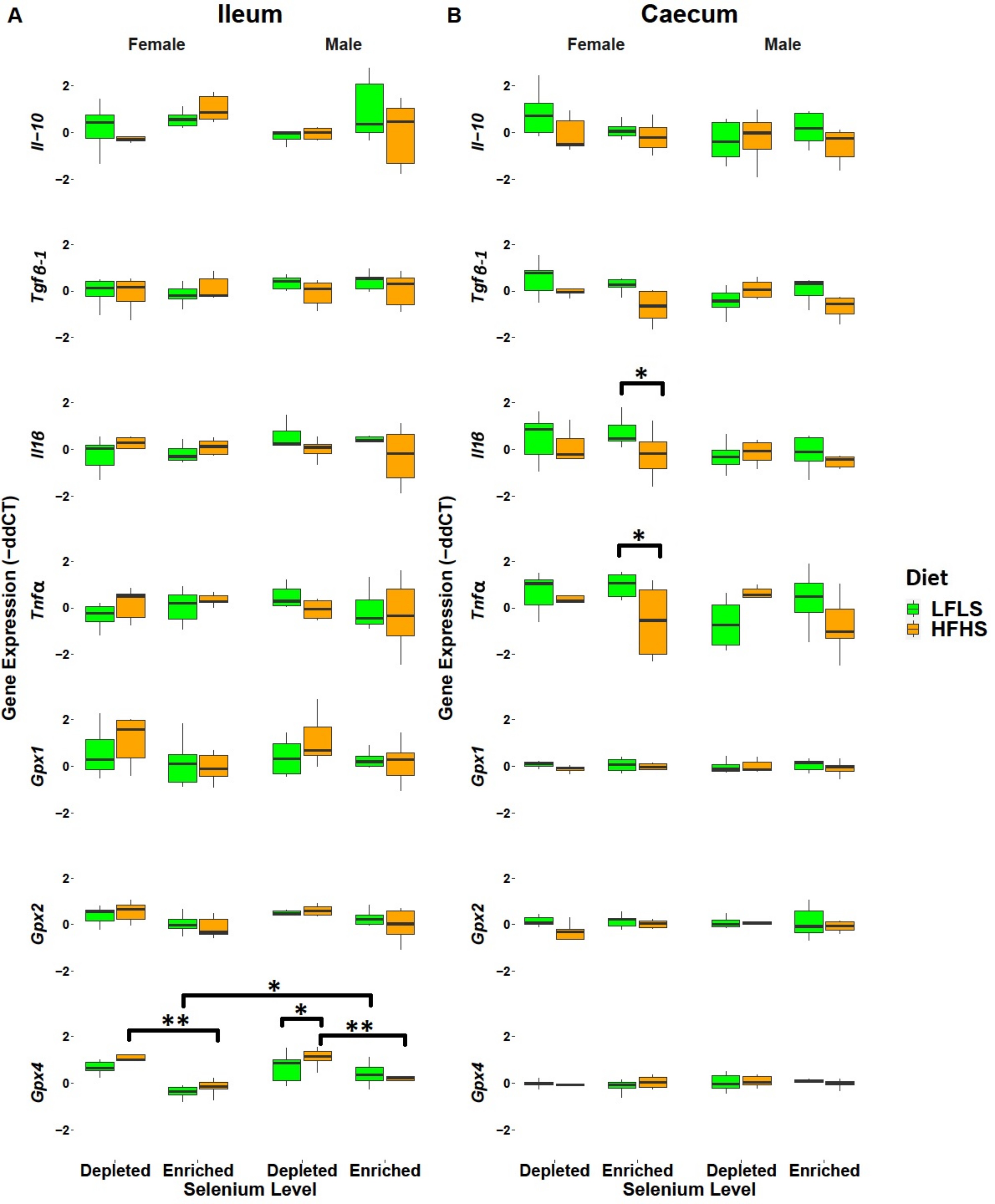

Supplement: Supplementary Figure 2 — Intestinal mRNA expression of immune response and anti-oxidant state genes as fold change (FC) calculated using the ΔΔCT method by sex. (A) Ileum. (B) Caecum. Gene expression was normalized to Hprt. P values of linear contrast analysis are detailed when significant ‘**’, P < 0.01, ‘*’, P<0.05 using contrast test between enriched and depleted Se levels, LFLS and HFHS formulations and the combination between Se levels and formulations. The samples were analysed at day 28 of the study. [file Image_2.jpeg]
